# Management of delayed cerebral ischemia after subarachnoid hemorrhage

**DOI:** 10.1186/s13054-016-1447-6

**Published:** 2016-10-14

**Authors:** Charles L. Francoeur, Stephan A. Mayer

**Affiliations:** 1 Critical Care Division, Department of Anesthesiology and Critical Care, CHU de Québec-Université Laval, Québec, Canada; 2 Department of Neurology (Neurocritical Care), Mount Sinai, New York, NY USA; 3 Institute for Critical Care Medicine, Icahn School of Medicine at Mount Sinai, One Gustave L. Levy Place, Box 1522, New York, NY 10029-6574 USA

**Keywords:** Delayed cerebral ischemia, Subarachnoid hemorrhage, Vasospasm, Multimodality monitoring

## Abstract

For patients who survive the initial bleeding event of a ruptured brain aneurysm, delayed cerebral ischemia (DCI) is one of the most important causes of mortality and poor neurological outcome. New insights in the last decade have led to an important paradigm shift in the understanding of DCI pathogenesis. Large-vessel cerebral vasospasm has been challenged as the sole causal mechanism; new hypotheses now focus on the early brain injury, microcirculatory dysfunction, impaired autoregulation, and spreading depolarization. Prevention of DCI primarily relies on nimodipine administration and optimization of blood volume and cardiac performance. Neurological monitoring is essential for early DCI detection and intervention. Serial clinical examination combined with intermittent transcranial Doppler ultrasonography and CT angiography (with or without perfusion) is the most commonly used monitoring paradigm, and usually suffices in good grade patients. By contrast, poor grade patients (WFNS grades 4 and 5) require more advanced monitoring because stupor and coma reduce sensitivity to the effects of ischemia. Greater reliance on CT perfusion imaging, continuous electroencephalography, and invasive brain multimodality monitoring are potential strategies to improve situational awareness as it relates to detecting DCI. Pharmacologically-induced hypertension combined with volume is the established first-line therapy for DCI; a good clinical response with reversal of the presenting deficit occurs in 70 % of patients. Medically refractory DCI, defined as failure to respond adequately to these measures, should trigger step-wise escalation of rescue therapy. Level 1 rescue therapy consists of cardiac output optimization, hemoglobin optimization, and endovascular intervention, including angioplasty and intra-arterial vasodilator infusion. In highly refractory cases, level 2 rescue therapies are also considered, none of which have been validated. This review provides an overview of current state-of-the-art care for DCI management.

## Background

Among subarachnoid hemorrhage (SAH) patients who survive the initial bleed of a ruptured aneurysm, delayed cerebral ischemia (DCI) is the most important preventable cause of mortality and poor neurological outcome. DCI affects up to 30 % of patients, and leaves the majority of survivors with motor deficits, cognitive dysfunction, and reduced quality of life [1]. The risk of DCI is primarily related to the severity of the initial hemorrhage, with a greater amount of cisternal and intraventricular blood on initial imaging and a poor post-resuscitation neurological examination being the strongest predictors of an unfavorable evolution [2].

State-of-the art management in the ICU does influence the outcome of DCI. In order to provide optimal care, clinicians must grasp the underlying concepts behind DCI and must all use the same terminology. Evidence-based interventions can be implemented to reduce the risk of developing DCI, adequate monitoring must be offered to allow early detection, and timely intervention should be offered to reverse DCI as rapidly as possible before the ischemic process progresses to infarction. We offer here a practical algorithm for managing DCI in the ICU based on the best available evidence, and on our expertise and experience in situations where firm data are lacking. The aim is to provide bedside clinicians with a structured and coherent approach in order to provide optimal care to their patients.

## Concepts and definitions

Historically, arterial narrowing with subsequent downstream low flow and ischemia was considered the sole cause of delayed neurological deterioration in SAH patients with vasospasm. This tenet of the SAH literature, however, has been challenged recently. Although the majority of SAH patients develop angiographic vasoconstriction (up to 70 %), only around 20–30 % develop DCI [2]. Cerebral infarction sometimes develops in the absence of demonstrable vasoconstriction, or in a vascular territory unaffected by vasospasm. Successful treatment of angiographic vasoconstriction does not necessarily lead to better functional outcome [3]. Clazosentan, an endothelin receptor antagonist, is successful in reducing angiographic vasospasm but has no significant effect on mortality, functional outcome, or the frequency of cerebral infarction [4]. Finally, nimodipine is the only pharmacological intervention shown to improve outcome in SAH patients, although it has no impact on large-vessel vasospasm [5].

Large artery vasospasm still doubtlessly plays an important role in the pathogenesis of DCI, but the scientific community has now turned its interest toward alternative explanations for a process that may be much more complex than was previously thought. The main thrust of this paradigm shift is general agreement that demonstration of large-vessel narrowing is no longer required to make the diagnosis of DCI. In line with recent publications and guidelines [6–8], we reserve the terms vasospasm for narrowing of large cerebral arteries as evidenced by imaging, DCI for cerebral infarction or neurological deterioration, or both, when the cause is thought to be vasospasm, and cerebral infarction as an infarct from any cause demonstrated on CT or MRI within 6 weeks of aneurysm rupture (see Table 1). The latter is now recognized as the primary determinant of long-term cognitive or motor deficits after SAH [9].

**Table 1:** Harmonized definition of delayed cerebral ischemia and cerebral infarction

**Delayed cerebral ischemia**
Focal (hemiparesis, aphasia, hemianopia, or neglect) or global (2 points decrease on GCS) neurological impairment lasting for at least 1 hour and/or cerebral infarction, which:
▪ Is not apparent immediately after aneurysm occlusion
▪ Is attributable to ischemia
▪ Is not attributed to other causes (i.e. surgical complication, metabolic derangements) after appropriate clinical, imaging, and laboratory evaluation
**Cerebral infarction**
Presence of cerebral infarction on CT or MR scan of the brain within 6 weeks after SAH, or on the latest CT or MR scan made before death within 6 weeks, or proven at autopsy; that is:
▪ Not present on the CT or MR scan between 24 and 48 hours after early aneurysm occlusion
▪ Not attributable to other causes such as surgical clipping or endovascular treatment
▪ Not due to a nonischemic lucency related to a ventricular catheter, intraparenchymal hematoma, or brain retraction injury

Based on references [101, 102]

*GCS* Glasgow Coma Scale, *CT* computed tomography, *MR* magnetic resonance, *SAH*, subarachnoid hemorrhage

## Pathogenesis

Although in-depth exploration of the pathophysiology of DCI is beyond the scope of this review, a basic understanding of the prevailing hypotheses is useful to the clinician. As mentioned earlier, large-vessel narrowing with subsequent low flow might be one of multiple mechanisms of DCI, but the causal framework now also includes early brain injury (EBI), microcirculatory dysfunction with loss of autoregulation, cortical spreading depolarization (CSD), and microthrombosis [10]. EBI encompasses the multiple physiological derangements that are thought to occur in the first 72 hours after the ictus. The initial ICP crisis and global hypoperfusion trigger glial activation, endothelial dysfunction, and inflammatory pathways. Animal and human data suggest an ultra-early diffuse neuroinflammatory process that predicts later ischemic damage [11]. Associated necrosis and apoptosis, as well as endothelial dysfunction, lead to neuronal loss and cerebral edema, respectively. CSD represents a wave of electrical depolarization that propagates across the cerebral gray matter at a speed of 2–5 mm/min, with ensuing depression of ECoG activity for 5–15 min [12]. This process is accompanied by neurovascular uncoupling: as the energy expenditure of neurons is reaching its peak, paradoxical vasoconstriction occurs, resulting in cortical hypoperfusion and energy failure. CSD is present in 80 % of poor grade SAH patients, has a biphasic distribution with peak frequency on SAH days 0 and 7, and has an uncertain relationship to large-vessel vasospasm and concurrent seizure activity [13]. Endothelial and platelet dysfunction, coagulation cascade activation, and impaired fibrinolysis all occur after SAH. Numerous biological markers of these events have been associated with DCI and poor outcome. Postmortem studies have found evidence of microthrombi, particularly in areas of cerebral infarction, after SAH. In fact, this correlates better with cerebral infarction lesions than vasospasm or aneurysm location [14].

## Prevention

### Nimodipine

DCI prevention has been the Holy Grail of SAH research for decades, but few options are available and unfortunately most attempts have yielded disappointing results (see Table 2). Nimodipine, a dihydropyridine calcium channel antagonist, is the only pharmacologic intervention so far associated with better outcome in SAH patients. Multiple trials have demonstrated a benefit [15], with the seminal trial showing an impressive reduction in cerebral infarction, poor neurological outcome, and death with oral nimodipine 60 mg given every 4 hours for 21 days [16]. This is now the recommended regimen, although intravenous nimodipine is approved as an alternative in Europe. Since nimodipine can cause hypotension, the dose can be divided into 30 mg every 2 hours or reduced to 30 mg every 4 hours. An ongoing phase 3 trial evaluating a single administration of intraventricular nimodipine (600 mg) microparticles to optimize its efficacy and reduce its side effects is in progress [17].

**Table 2:** Selected pharmacologic interventions that have been evaluated for DCI prevention^a^

Intervention	Effect
Aspirin	No effect on new lesion associated with neurological worsening [103]
Clazosentan	No effect on mortality or vasospasm-related morbidity [5]
Enoxaparin	No effect on DCI or GOS at 3 months [104]
Erythropoietin	Less neurological deficit with cerebral infarct; no difference in mRS or GOS at 6 months [105]
Fludrocortisone	No effect on incidence of cerebral ischemia or independent living [27]
Magnesium	No difference in mRS at 3 months [106]
Methylprednisolone	No effect on neurologic worsening; trend towards better GOS at 6 months [107]
Nicardipine	No effect on neurological worsening or GOS at 3 months [102]
Prophylactic angioplasty	No effect on new neurologic deficits or GOS at 3 months [86]
Prophylactic hypervolemia	No effect on neurologic worsening or GOS at 3 months [69]
Statins	No effect on DCI, death or mRS at 6 months [108]

^a^Excluding nimodipine. Only randomized controlled trials are considered. References are either the most recent, most definitive, or most robust trial according to the authors’ opinion

*DCI* delayed cerebral ischemia, *GOS*, Glasgow Outcome Scale, *mRS* modified Rankin Scale

### Enhanced blood clearance

The presence of blood and its breakdown products is strongly associated with vasospasm. Numerous attempts have been made to accelerate clearance of subarachnoid blood, with the hope that this might result in the prevention of delayed arterial spasm. The only randomized controlled trial (RCT) investigating the use of intraoperative administration of rt-PA failed to show any effect on outcome [18]. Lumbar drainage of CSF was also unsuccessful at improving mRS [19] or GOS [20] scores at 6 months in two RCTs. Different other interventions, including cisternal irrigation or use of urokinase, have been evaluated for feasibility and reported mixed results. Use of such techniques cannot be advocated at present.

### Avoidance of hypovolemia and hyponatremia

Hyponatremia and hypovolemia occur frequently after SAH due to physiological changes favoring excessive natriuresis and inappropriate antidiuretic hormone elevation, and have been associated with impending DCI [21]. Retrospective data indicate that fluid restriction, the typical treatment for syndrome of inappropriate antidiuretic hormone (SIADH), can be deleterious and increases the risk of DCI due to aggravation of hypovolemia [22]. Isotonic crystalloid fluid resuscitation targeting normal serum sodium values and euvolemia is presently the favored fluid management strategy for preventing DCI. The latter is notoriously difficult to assess in critically ill patients, and the readers are referred to papers dedicated to this specific subject for a more in-depth approach to the matter [23–26]. Administration of fludrocortisone (between 0.2 and 0.4 mg/day) has been shown to reduce the occurrence of hyponatremia [27], with some indication towards DCI reduction. Anecdotal evidence indicates that correction of acute symptomatic hyponatremia with hypertonic saline (3 %) infusion is usually effective.

## Detection and diagnosis

Early detection of DCI is critical to allow for timely intervention. Although straightforward in relatively intact patients, early detection is notoriously difficult in poor grade SAH patients (Table 3). Depending on the context, the technique can vary from simple serial clinical examinations to multiple advanced monitoring strategies, as described in the following section.

**Table 3:** Components of brain multimodality monitoring for poor grade SAH

Device	Physiological parameter measured	Normal range	Pathological condition
Continuous electroencephalography	Brain activity Epileptiform discharges	• Alpha/delta ratio > 50 % • No epileptiform discharges • Reactivity to stimuli	• Alpha/delta ratio < 50 % • Epileptiform discharges • No reactivity
Transcranial Doppler ultrasound	Mean blood flow velocity (FVm)	• FVm MCA: 30–75 cm/s	• MCA FVm 120–180 cm/s: intermediate probability of vasospasm • MCA FVm >180 cm/s: high probability of vasospasm
Cerebral blood flow monitor (Hemedex)	Cerebral blood flow (CBF)	• >40 ml/100 g/min	• <20 ml/100 g/min: indicative of ischemia assuming preserved metabolic demand
Jugular venous oximetry	Balance between oxygen delivery and consumption (SjO_2_)	• 50–75 %	• <50 %; increased oxygen extraction fraction, indicative of ischemia
Brain tissue oxygen tension (Licox)	Regional parenchymal brain tissue oxygen tension (PbtO2)	• 25–35 mmHg in white subcortical matter	• <20 mmHg: indicative of cerebral hypoxia
Cerebral microdialysis	• Glucose • Lactate • Pyruvate • Lactate/pyruvate ratio • Glutamate • Glycerol	• 0.8–4.0 μmol/L • 0.7–3.0 μmol/L • Unknown • < 25 • 2–10 μmol/L • 10–90 μmol/L	• <0.2 μmol/L • ≥4.0 μmol/L • Unknown • >40 indicative of anaerobic metabolism • >10 μmol/L • >90 μmol/L

*SAH* subarachnoid hemorrhage

### Clinical examination

Clinical examination in awake patients who can follow commands is the most reliable way to detect and diagnose DCI. Neurological impairment can be focal or global. The Glasgow Coma Scale (GCS) is the most commonly used tool for measuring and documenting the level of consciousness in the ICU setting. Serial testing of attention and concentration by reciting from 20 to 1 and from December to January in good grade patents has been used successfully to quantify subtle changes in mental status that are not detected by the GCS [28]. However, poor grade SAH patients, defined here as WFNS grades 4 and 5, do not consistently manifest symptoms when DCI occurs, although they constitute the most at-risk group. More than 20 % will present DCI as asymptomatic cerebral infarction, and these patients are less likely to receive acute hypertensive therapy [29]. This is the primary rationale for using other modalities, including invasive brain multimodality monitoring (MMM) [30], in this specific subgroup.

### Transcranial Doppler ultrasonography

Transcranial Doppler (TCD) ultrasonography is a noninvasive test that allows indirect detection of large-vessel narrowing based on quantification of acceleration of flow. Velocities lower than 120 cm/s in the middle cerebral artery (MCA) show high negative predictive value for angiographic vasospasm, whereas velocities exceeding 180 cm/s have high positive predictive value [31]. The Lindegaard ratio, defined as MCA mean cerebral blood flow (CBF) velocity divided by extracranial internal carotid artery mean cerebral flow velocity, is an index thought to be less affected by systemic hemodynamic variations. Used as a screening tool in many tertiary centers, TCD ultrasonography suffers from both technical and anatomical limitations [32]. TCD ultrasonography provides no information about the distal vasculature and can be affected by hydrocephalus or elevated intracranial pressure. Proper vessel insonation is highly operator dependent and at least 10 % of patients do not have adequate bone windows. Finally, just as with vascular imaging, TCD ultrasonography detects vasospasm, but this does not directly translate into a high risk of DCI. In one study, 40 % of SAH patients who experienced DCI never had a MCA flow velocity that exceeded 120 cm/s during the entire period of monitoring [33]. It is the authors’ opinion that the aforementioned cutoff values are specific enough to mandate additional investigations if the clinical picture is compatible with impending or ongoing DCI. However, due to its low sensitivity, TCD ultrasonography should not be the sole screening examination in a patient with a poor clinical examination.

### Vascular imaging

Imaging of the cerebral vasculature allows recognition of arterial narrowing. A decrease in luminal diameter of more than 50 % is usually considered severe vasospasm and is associated with lower CBF. Conventional angiography (digital subtraction angiography (DSA)) is the gold standard and offers the possibility of endovascular treatment. Complication rates for diagnostic angiography are in the range of 1 %. Computed tomographic angiography (CTA) is a less invasive and a more readily available option. Studies comparing CTA with DSA have found good agreement, suggesting high sensitivity and specificity in vasospasm diagnosis [34]. The authors use CTA as a first-line screening tool for detecting large-vessel vasospasm, with the initial study timed to occur between SAH day 4 (for patients felt to be at greater risk) and day 8 (for lower risk patients). Lack of appreciable large-vessel spasm on SAH day 8 or later implies a very low risk of subsequent DCI, enabling fast-tracking out of the ICU into a lower intensity, step-down setting.

### Brain perfusion imaging

**Fig. 1:**
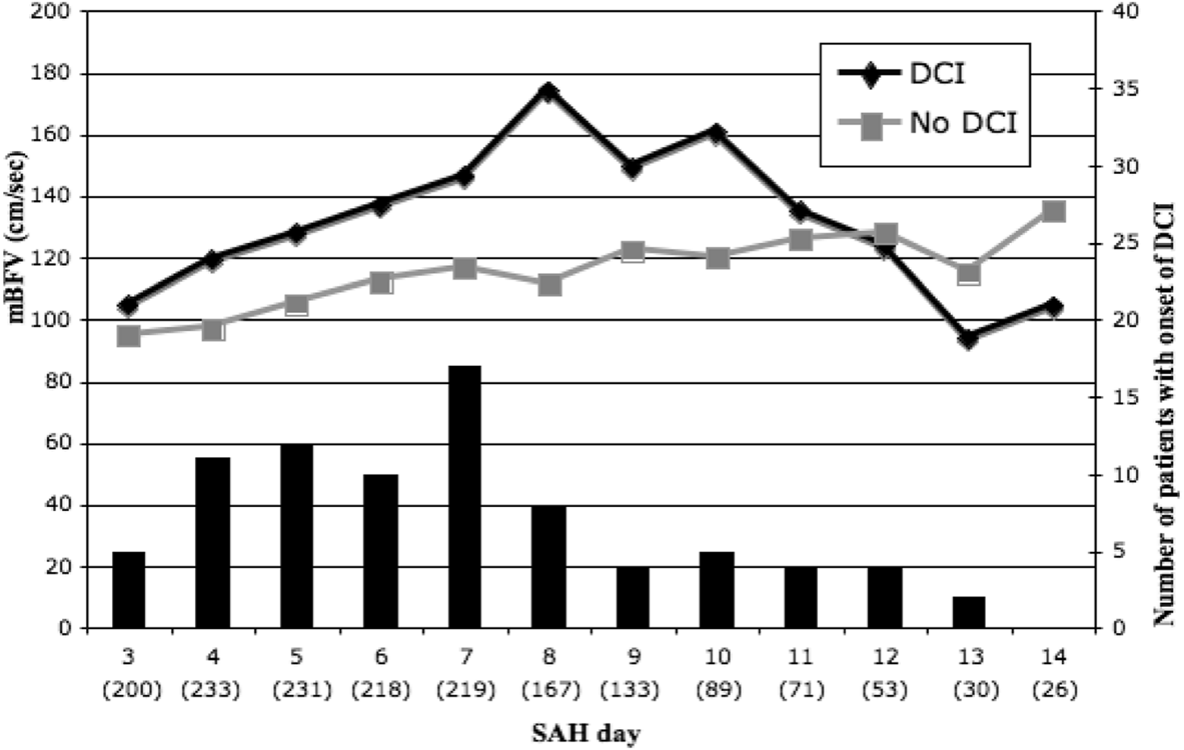
Mean maximal TCD values during SAH days 3–14 in patients who did or did not develop DCI. TCD examinations after the diagnosis of DCI were censored. Histogram shows the number of patients with new onset DCI between SAH days 3 and 14. Nine patients had DCI between days 15 and 29. Number (*in parentheses*) represents the number of TCD examinations performed for each corresponding SAH day. From reference [33], with permission. *DCI* delayed cerebral ischemia, *mBFV* mean blood flow velocity, *SAH* subarachnoid hemorrhage

Directly assessing cerebral perfusion is appealing because it allows for evaluation of the functional consequences of both large-vessel and small-vessel vasospasm. Xenon CT, single photon emission computed tomography, positron emission tomography, MR perfusion, and computed tomographic perfusion (CTP) all allow tomographic CBF assessment. CTP is currently the most widely used and studied modality [35]. Various cutoff values that correlate with DCI have been reported, including a mean transit time (MTT) exceeding 5.0–6.4 s, or regional CBF below 25–40 ml/100 g/min [36]. One detriment to this type of analysis is the high degree of variability due to differences in equipment and postprocessing methods [37]. CTP seems to correlate fairly well with DCI, but focal flow reductions can also occur as a consequence of brain retraction injury or perihematomal brain dysfunction. Many centers perform CTA and CTP together, as a complement to serial TCD monitoring, in the critical time window for DCI onset (SAH days 4–8, see Fig. 1).

### Continuous electroencephalography

Continuous electroencephalography provides noninvasive, real-time continuous information about cortical activity, and quantitative electroencephalography allows decomposition of the data contained in the raw EEG. In the presence of cortical hypoperfusion leading to neuronal dysfunction, EEG changes are detectable and may precede the onset of symptoms [38]. Recent data suggest that reductions in the alpha/delta ratio (ADR) or in alpha variability are most sensitive and specific for predicting DCI at a point where it is potentially reversible [39]. Even more interesting, reversal of those changes could serve as a surrogate target to titrate therapy. For example, as explained later, induced hypertension could be titrated to ADR normalization. Despite its theoretical attractiveness, the intense manpower commitment required to provide around-the-clock real-time neurotelemetry has hampered widespread adoption of continuous electroencephalography for neuromonitoring after SAH.

### Multimodality monitoring

Advanced neuromonitoring using MMM provides continuous, real-time information allowing early detection of physiological derangements, providing both a trigger and a target for intervention. In addition to acting as an early warning system for improving situational awareness, MMM can be proactively used to create an optimized physiological environment for the injured brain, with the goal of secondary injury prevention. Many high-volume centers equipped with invasive MMM now routinely use it in poor grade SAH patients, with various combinations of ICP, brain tissue oxygen, CBF, and metabolic monitoring, as well as intracranial electroencephalography.

ICP monitoring is essential to any MMM bundle. Intracranial hypertension is common in SAH, especially in poor grade patients where occurrence in up to 80 % of patients has been described [40]. It is associated with severely deranged cerebral metabolism [41] and consistently leads to poor outcome [42, 43], warranting aggressive management. ICP monitoring also permits cerebral perfusion pressure (CPP) measurement. We have reported in poor grade patients that simply maintaining CPP >70 mmHg is associated with a lower risk of brain metabolic crisis and tissue hypoxia [44], which may be a useful clinical guideline for minimizing the risk of secondary brain injury in unmonitored patients.

Parenchymal brain tissue oxygenation (PbtO2) monitoring allows quantification of oxygen tension in the brain interstitial space and will detect episodes of cerebral compromise even in the absence of elevated ICP or low CPP [30], underlying its role as a complement to conventional neuromonitoring in SAH patients. This is probably helpful in early detection of silent infarcts [29], and higher mean PbtO2 is associated with improved survival [30].

Microdialysis allows determination of interstitial fluid composition and cellular metabolism. The most common targets of clinical microdialysis analysis are extracellular lactate levels and the lactate/pyruvate ratio (LPR) [45]. These metabolic derangements precede silent infarction by a few hours [29], are often detected in the setting of normal ICP and even normal PbtO_2_ [30], and are fairly specific for DCI (0.89 for lactate levels > 4 mmol) [46]. Microdialysis is actually superior to TCD ultrasonography and DSA in predicting clinical deterioration secondary to DCI [47]. Some experienced centers also use the biochemical profile to differentiate ischemia from mitochondrial dysfunction [48] or to monitor brain glucose metabolism [49], but these applications need further evaluation before being widely adopted.

Intracranial electroencephalography includes subcortical electrocorticography (ECoG) and intracortical electroencephalography (ICE). ECoG allows detection of CSD ischemia, a potent mechanism of DCI [13] that decreases brain O_2_ supply and increases brain O_2_ consumption in SAH patients [50], providing a potential therapeutic target [51]. ICE, on the other hand, can detect ictal discharges not apparent on scalp EEG [52]; ICE ADR reduction may outperform scalp quantitative electroencephalography in early DCI detection [53].

Finally, ICP or PbtO_2_ monitoring also permits dynamic evaluation of autoregulation through moving linear correlation coefficients such as the pressure reactivity index (PRx, which correlates MAP with ICP) or the PtiO_2_ pressure reactivity index (ORx, which correlates PbtO_2_ with CPP) [54]. Early autoregulatory failure is predictive of DCI [55] and is associated with poor outcome in SAH patients [56]. Theoretically, these indices might also be used to define the optimal CPP for a given patient [57].

Proper positioning in at risk cerebral region is essential, but offers no guarantee that other brain regions are not ischemic [58]. We prefer to place the MMM bolt in the frontal anterior and middle cerebral territory watershed region ipsilateral to the ruptured aneurysm, or in the nondominant hemisphere in the case of a midline aneurysm. The invasive and regional nature of MMM, its associated cost, and the required expertise are the main obstacles to its implementation.

## Treatment

**Fig. 2:**
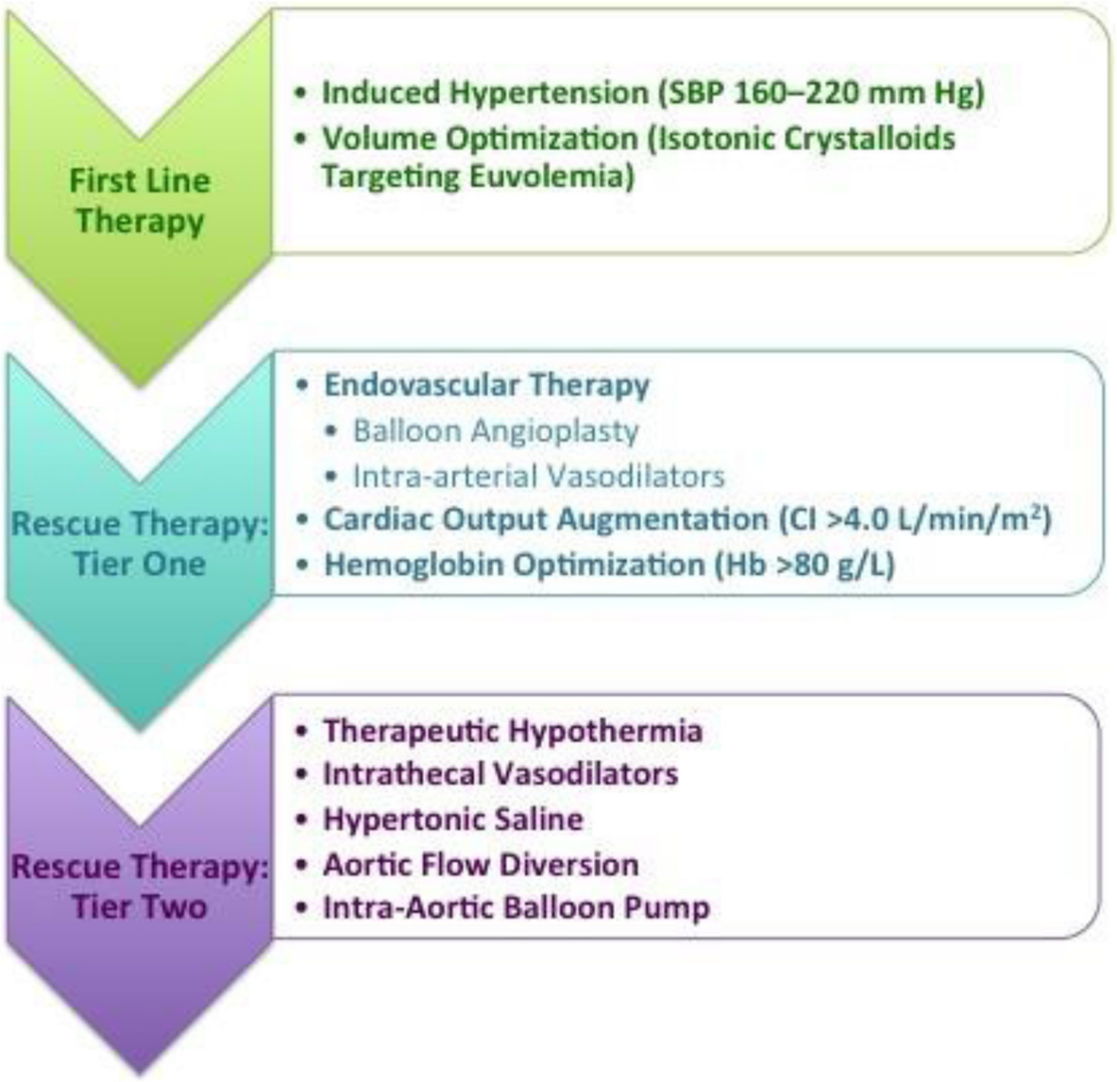
Stepwise approach to the treatment of active DCI from vasospasm. The order or the intensity of therapy must be adapted to each situation. *CI* cardiac index, *Hb* hemoglobin, *SBP* systolic blood pressure

SAH patients are complex and should be cared for in specialized, high-volume centers to maximize good outcome [59]. The suggested approach below assumes that the standards of care in all other aspects of treatment are followed. An organized approach that has been agreed upon in advance by all stakeholders minimizes conflicts and streamlines the process of care. Although presented as a three-stage algorithm (Fig. 2), management should always be tailored to the individual patient, to the available resources, and in a contextualized fashion. Our approach to treatment divides interventions into: first-line therapy for new-onset DCI, which can manifest as neurological deterioration, characteristic imaging findings, or MMM abnormalities indicative of ischemia; and second-line “rescue therapy” for refractory DCI, indicating inadequate reversal of ischemia in response to first-line therapy.

### First-line therapy for new-onset DCI

#### Induced hypertension

Successful reversal of neurological symptoms following induced hypertension has been described in case series since the late 1970s, and most clinicians caring for SAH patients can testify to its benefit. Use of vasopressors to augment blood pressure is still the cornerstone of first-line therapy for DCI. A normal saline bolus (15 ml/kg over 1 hour) at the institution of therapy increases CBF [60]. Norepinephrine [61], dopamine [62], and phenylephrine-induced [63] hypertension all have been demonstrated to significantly improve CBF and/or cerebral oxygenation, resulting in clinical improvement of the neurological deficit in approximately 70 % of patients. The authors use norepinephrine as the first-line treatment of choice due to its combination of alpha and beta receptor stimulation, the low frequency of tachycardia, and the reliable hemodynamic response that results. Arginine vasopressin has also been reported as a safe supplementary vasopressor in a small group of SAH patients [64]. We reserve its use for refractory DCI patients when multiple vasoactive agents are required to attain hemodynamic targets.

A starting systolic target ranging between 160 and 180 mmHg is usually selected, depending on the patient’s baseline blood pressure. Mean arterial pressure (MAP) can be used as an alternative to systolic pressure, as per unit standards. In poor grade patients with an ICP monitor, induced hypertension should be targeted at increasing CPP, which is the relevant perfusing pressure of the brain. The target can then be increased stepwise in a goal-directed fashion and titrated to clinical response, which is usually linked to what triggered intervention in the first place. In symptomatic patients with a reliable clinical examination, the goal is resolution of symptoms. In poor grade patients, clinicians must rely on available monitoring, including reversal of changes in PbtO_2_, LPR, and continuous electroencephalography. Once therapy is instituted, absence of response in 30 min should trigger an escalation of the BP target. Most centers use a maximal target range of around 120 mmHg for CPP, 140 mmHg for MAP, and 220 mmHg for SBP. Clinicians should monitor for complications such as heart failure and myocardial demand ischemia. Recent data confirm that pursuing induced hypertension in patients with unruptured, unsecured aneurysms is safe [65].

As far as de-escalation of hypertensive therapy is concerned, the literature is devoid of guidelines. The authors obtain at least a 24–48-hour window of stable neurological condition before deescalating in a stepwise fashion, monitoring for recurrence of ischemia. While induced hypertension is now hardwired in clinical practice and in every guideline, its impact on outcome has not yet been submitted to the scrutiny of a RCT. This was the aim of the HIMALAIA study (Hypertension Induction in the Management of AneurysmaL subArachnoid haemorrhage with secondary IschaemiA) [66], a multicenter RCT that was terminated in 2015 due to slow recruitment. This termination confirms that it seems unlikely any such trial will ever be conducted given the lack of clinical equipoise.

#### Volume optimization

As induced hypertension was embraced as a therapy for symptomatic DCI in the 1980s, the concept of hemodynamic augmentation for DCI evolved into a bundle of hypertension, hypervolemia, and hemodilution: the so-called “Triple-H” therapy [67, 68]. It has since become apparent that the hypervolemia component is probably useless and might actually be harmful [61]. In one clinical trial, prophylactic hypervolemic therapy directed toward maintaining elevated central venous pressure failed to prevent DCI; the additional volume resulted in no net increase in cumulative fluid balance, blood volume, or CBF [69]. Other studies have shown that hypervolemic therapy increases the risk of pulmonary edema, especially in the setting of cardiac dysfunction [70], and that positive fluid balance in SAH is associated with poor outcome [71]. Current guidelines suggest that isotonic fluids be used judiciously to correct hypovolemia, with the ultimate goal of maintaining a euvolemic state while avoiding fluid overload [7].

### Rescue therapy for medically-refractory DCI

#### Tier One interventions

##### Hemoglobin optimization

Based on current evidence from randomized clinical trials in the general ICU population [72], a restrictive strategy aiming for a hemoglobin level above 70 g/L is the favored approach for SAH patients prior to the onset of DCI. It is questionable, however, whether this is the appropriate threshold for patients with active and ongoing brain ischemia. Anemia is seen in more than 50 % of SAH patients [73] and is consistently associated with poor outcome [74, 75]. Moreover, hemoglobin levels of less than 90 g/L, and even less than 100 g/L, are associated with brain tissue hypoxia and metabolic distress in poor grade patients [76]. Packed red blood cell transfusion successfully increases brain tissue oxygen tension in poor grade SAH patients with a baseline hemoglobin level of 80 g/L [77]. This makes the use of red blood cell transfusion to optimize cerebral oxygen delivery appealing when facing active brain ischemia refractory to first-line therapies. However, blood transfusions are also associated with medical complications [78], poor outcome [79], and even higher mortality in the [80] SAH population. The ongoing RCT Aneurysmal Subarachnoid Hemorrhage: Red Blood Cell Transfusion and Outcome (SAHaRA Pilot) comparing RBC transfusion triggers from 100 g/L down to 80 g/L will hopefully shed light on this debate. In the meantime, the Neurocritical Care Society guidelines [7] suggest a transfusion threshold of 80 g/L in SAH patients without DCI, with a more aggressive transfusion trigger of 90–100 g/L as a Tier One rescue therapy in cases of DCI unresponsive to first-line therapy.

##### Endovascular therapy

When confronted with medically refractory DCI—cases in which significant neurologic deficits exist despite hemodynamic optimization—endovascular treatment should be the next step [81]. In recent years, indications for deploying intra-arterial therapy have evolved and this treatment is introduced much earlier, especially if there are reasons to believe that medical therapy is at high risk of failure or in the face of complications resulting from heart failure, fluid overload, or myocardial ischemia [82].

Endovascular therapy can be subdivided into mechanical dilation and intra-arterial infusion of vasodilators. Percutaneous transluminal balloon angioplasty (PTCA) is based on mechanical stretching and dilation of vasospastic arteries. The technique is limited to proximal vessels, mainly the internal carotid artery and vertebral or basilar artery, M1 and sometimes M2 segments of the MCA, and A1 and P1 segments of the anterior and posterior cerebral artery respectively. The success rate in most case series is over 90 % and long-lasting [83], with occasional cases of recurrence that require repeated procedures. Improvement in CBF post PTCA has also been clearly demonstrated [84]. Observational studies suggest that early intervention (less than 2 hours after neurological decline) results in a better clinical response [85]. The drawback of PTCA is that serious complications can occur in up to 5 % of patients, including embolism, thrombosis, dissection, and vessel rupture. The only published RCT to date evaluated PTCA as a prophylactic measure in good grade patients with large amounts of cisternal clot [86]. Three patients died of vessel perforation and there was no difference in frequency of DCI, condemning this indication. If the clinician is convinced that ongoing ischemia is explained by the visualized local vasospasm, PTCA is a potent therapy.

Numerous cases series have shown various degrees of reversal of vasospasm with intra-arterial vasodilators, evaluated by angiography, TCD ultrasonography, Xenon CBF, cerebral oxygenation, or angiographic cerebral circulation time. Over the years, numerous agents have been evaluated, including papaverine, nicardipine, verapamil, nimodipine, milrinone, amrinone, and fasudil. None of these have ever been tested objectively in a clinical trial against a control group. Intra-arterial vasodilators have several advantages over PTCA: better distal penetration, a more diffuse effect, and a better safety profile. It is most often used with balloon angioplasty, for more distal or diffuse vasospasm. Disadvantages include recurrent vasospasm due to the short-lasting effect of these agents, increased ICP secondary to vasodilation [87], and potential hypotension due to systemic effects. Today the most commonly used agents are intra-arterial nicardipine 10–20 mg or verapamil 20–40 mg, infused over about 1 hour. Doses of up to 720 mg per treatment have been described in refractory severe vasospasm [88].

##### Cardiac output augmentation

Several authors have demonstrated that increasing cardiac output (CO) with fluids and inotropes is feasible and can improve brain perfusion after SAH [89]. CO augmentation with dobutamine has been shown to increase CBF by almost 50 % in SAH patients with severe vasospasm, which is comparable with the effect of phenylephrine [63]. Milrinone, a selective inhibitor of the phosphodiesterase III isoenzyme, provides more effective inotropy than dobutamine in the setting of neurogenic stunned myocardium, which is associated with beta-receptor desensitization [90]. The Montreal Neurological Institute published an uncontrolled case series in which high-dose milrinone (0.75–1.25 μg/kg/min) was used as first-line therapy with good results, without CO monitoring, to improve microcirculatory flow [91]. By contrast, the authors and most centers use CO augmentation as a second-line hemodynamic intervention once arterial BP has been optimized. The authors recommend the use of a validated CO monitoring device, such as a transpulmonary thermodilution (PICCO; Maquet Medical) or a pulmonary artery catheter, to titrate fluids, pressors, and inotropes [92], targeting a cardiac index of >4.0 L/min/m^2^.

#### Tier Two interventions

When facing evidence of ongoing neurological injury despite the aforementioned measures, the clinician is left with the option of pursuing nonevidence-based therapies. These interventions should only be instituted in centers with the appropriate expertise and monitoring, and should be proportionate to the global goals of care. Infusion of hypertonic saline (2 ml/kg of HTS 23.5 % over 20 min) has been shown to improve CBF [93] in poor grade patients and can be considered if facing elevated ICP concomitantly to DCI. Most clinicians will favor advanced fever control, even if it requires heavier sedation or paralysis [94]. The next step involves targeted temperature management to attain hypothermia to levels between 33 and 36 °C [95], with or without use of barbiturates [96]. Although this has been described, no objective substantiation of success or safety is available.

Experimental interventions include aortic flow diversion, intrathecal nicardipine, and intra-aortic balloon pump (IABP) counter-pulsation. The aortic flow diversion NeuroFlo System™ (Zoll Medical) partially occludes the descending aorta in order to divert a greater proportion of the CO towards the brain, resulting in higher perfusion pressure and microcirculatory flow [97]. Its use remains investigational and in the USA is limited to a Food and Drug Administration Humanitarian Device Exemption. Intrathecal nicardipine, given via a ventricular catheter, has been reported to reduce TCD velocities within 8 hours of administration and has been used off-label as rescue therapy for patients with refractory DCI [98]. Intrathecal nitroprusside has also been evaluated as a potential therapy for refractory vasospasm [99]. Finally, anecdotal reports mention successful use of an IABP in cases of refractory DCI associated with severe cardiac dysfunction, making it another option to consider in extreme cases [100].

## Conclusion

DCI prevention, detection, and reversal are among the top priorities of clinicians caring for SAH patients. Based on the best available evidence, nimodipine administration and maintenance of euvolemia are the surest way to prevent DCI. Detection of delayed ischemia can rely on simple clinical examination in intact patients, but requires advanced MMM in poor grade patients. Early diagnosis and treatment is the key to treating active, symptomatic DCI. Induced hypertension and volume optimization are the cornerstone of first-line therapy. Rescue therapy for medically refractory vasospasm relies primarily on endovascular intervention and circulatory optimization. A shift from the paradigm emphasizing large-vessel narrowing to recognition that vasospasm represents a complex, multifaceted pathophysiological process involving the microcirculation, disturbed autoregulation, and spreading depolarization should allow for new insights and novel therapeutic targets in the future. Fast-paced developments in imaging and advanced neuromonitoring also promise better understanding and earlier detection of DCI. Although fraught with many difficulties, dogma not being the least of them, new interventions will have to face rigorous trials in order to move towards a better outcome for our patients.

## Abbreviations

ADR: Alpha/delta ratio
CBF: Cerebral blood flow
CO: Cardiac output
CPP: Cerebral perfusion pressure
CSD: Cortical spreading depolarization
CTA: Computed tomographic angiography
CTP: Computed tomographic perfusion
DCI: Delayed cerebral ischemia
DSA: Digital subtraction angiography
EBI: Early brain injury
ECoG: Electrocorticography
EEG: Electroencephalogram
GCS: Glasgow Coma Scale
ICE: Intracortical electroencephalography
ICP: Intracerebral pressure
MAP: Mean arterial blood pressure
MCA: Middle cerebral artery
MMM: Multimodality monitoring
PTCA: Percutaneous transluminal balloon angioplasty
RCT: Randomized controlled trial
SAH: Subarachnoid hemorrhage
SBP: Systolic blood pressure
TCD: Transcranial Doppler
WFNSS: World Federation of Neurological Surgeons Scale
